# Reply to Offiah et al.: The triad of shaken baby syndrome

**DOI:** 10.1007/s00247-017-3858-1

**Published:** 2017-04-22

**Authors:** E. Susanna Axelsson, Jan O. Liliemark, Sofia H. Tranaeus

**Affiliations:** Swedish Agency for Health Technology Assessment and Assessment of Social Services, P.O. Box 6183, SE-102 33 Stockholm, Sweden

Dear Editor,

In response to Offiah et al. [1], the Swedish Agency for Health Technology Assessment and Assessment of Social Services is tasked by the Swedish government to evaluate methods used in the areas of health care and social services. Our evaluations are intended to provide guidance to practitioners as well as to political and administrative decision makers throughout Sweden. Our report is the result of a careful systematic evaluation of all of the available scientific evidence by a team that includes several experts in relevant scientific areas. For detailed information on our scientific method, please consult our methodology handbook, www.sbu.se/globalassets/eng_metodboken_160808.pdf. The report has no legal status. It is a scientific health technology assessment. Therefore, it is not expected to have any direct legal impact.

It is not customary to use peer review by specialist organisations in other countries for governmental investigations. This report has been extensively reviewed by our internal quality assurance board and by external experts, and has been carefully scrutinised by our board of directors as well as our scientific advisory boards. Therefore, additional international peer review would have been unlikely to provide relevant new information to change the report’s conclusions and motivate the considerable delay.

The English version of this report is published on our website: http://www.sbu.se/en/publications/sbu-assesses/traumatic-shaking--the-role-of-the-triad-in-medical-investigations-of-suspected-traumatic-shaking/.
